# Empowering young minds through STEM education: Engaging high schoolers in Ghana through medical physics

**DOI:** 10.1002/acm2.70126

**Published:** 2025-05-13

**Authors:** Afua A. Yorke, Mercy T. Schandorf, Abigail N. M. Quaye, Peniel Tenkorama Twum, Bishwambhar Sengupta, Kwadwo Nkansah‐Poku, Juliana A. Kplorfia, Jessica Fagestrom

**Affiliations:** ^1^ University of Washington Fred Hutch Cancer Center Seattle Washington USA; ^2^ University of Ghana Medical Center Accra Ghana; ^3^ Komfo Anokye Teaching Hospital Oncology Directorate Kumasi Ghana; ^4^ Global Communities Accra Ghana; ^5^ University of Ghana Medical School Accra Ghana; ^6^ Girls Excellence Movement Accra Ghana

**Keywords:** education, international outreach, radiation therapy, student engagement, teaching

## Abstract

**Purpose:**

To promote diversity in Science, Technology, Engineering, and Mathematics (STEM), an educational presentation and hands‐on session was organised to raise awareness of STEM career opportunities among high school girls to introduce the students to the field of medical physics.

**Materials and Methods:**

The study involved 65 first‐year Senior High School girls, aged 13–16, pursuing general science in Accra, Ghana. This initiative, organised by the Girls Excellence Movement (GEM) in collaboration with a United States (US) institution, implemented the “*heroes in radiation oncology*” program, which included a relatable presentation and hands‐on experience in simulation to treatment planning activities. The program's effectiveness was assessed through pre‐and post‐assessment surveys, and a thematic analysis of student feedback.

**Results:**

Participants' awareness of career fields showed an interest in traditional healthcare professions (92%) and engineering (73.8%), with minimal medical physics awareness (12.3%). Post‐presentation survey showed a significant change in participants' perception of medical physics 87.3%. Thematic analysis revealed increased awareness, understanding, and interest, dispelled misconceptions about radiation safety, and highlighted the interdisciplinary nature and career opportunities. The presentation was successful in inspiring participants and expanding their perspectives on medical physics.

**Conclusion:**

The program raised awareness of medical physics among participants, many of whom were previously unfamiliar with the field. Participants reported a newfound understanding of the interdisciplinary nature of medical physics, its connections to biology, mathematics, and engineering.This program can easily be reproduced in community and school outreaches.

## 1 INTRODUCTION

In many low‐income countries, young students, particularly girls, have limited exposure to the Science, Technology, Engineering, and Mathematics (STEM) fields.

The importance of equal opportunities for women in STEM education in Africa cannot be overlooked. The United Nations Education, Scientific and Cultural Organisation (UNESCO) reports that women constitute only 28% of researchers globally,^1^, ^2^, ^3^, ^4^, ^5^ with significant disparities in Africa due to cultural and social barriers.^1^, ^3^, ^6^, ^7^, ^8^ A UNESCO Institute for Statistics (UIS) report reveals that, in Ghana, about 18.3% of women in science are researchers.^1^ Key factors influencing these trends include societal norms and systemic barriers that hinder access to scientific careers for women and girls.^2^, ^3^, ^6^, ^7^, ^8^, ^9^, ^10^, ^11^, ^12^ UNESCO also found that a girl's interest in STEM is closely linked to her perception of self‐efficacy and is shaped by her social environment,^10^, ^12^ including parental expectations,^13^ peer influence,^10^, ^11^, ^14^ stereotype threat^15^, ^16^, ^17^ and media representation.^18^ Additionally, early learning experiences,^19^ teaching strategies,^20^, ^21^ curriculum,^22^ and opportunities for mentorship play a crucial role in fostering interest in STEM among girls.^23^, ^24^

Despite its vast human potential and resources, Africa faces a significant disparity in STEM education, representing a missed opportunity. Strategic interventions can bridge this gap, fostering equal opportunities for African youth and driving sustainable economic development. Research by Levine et al.^6^ and Wang & Degol^9^ indicates that awareness of STEM opportunities enhances interest in these fields. To address the underrepresentation of African girls and women in STEM, various international and local organizations are implementing initiatives to improve their participation and success in STEM education. The Mastercard Foundation collaborates with local partners to create initiatives that enhance participation, including Leaders in Teaching, the African Institute for Mathematical Sciences (AIMS), and scholarship programs.^25^ Other organizations in Africa and Ghana promote women in STEM through programs like coding clubs. Notable groups involved include the Girls Excellence Movement (GEM),^26^ Women in Nuclear‐Global (WiN),^27^ The Organization for Women in Science for the Developing World (OWSD),^28^ African Women in Agricultural Research and Development (AWARD),^29^ and Women in Science Technology Engineering and Mathematics (WiSTEM).^30^

Various organizations have hosted events over the years to foster an interest in STEM among senior high school students while also addressing misconceptions and biases associated with these fields. The Ghana chapter of the African Women in Agricultural Research and Development (GhaWARD) has organized numerous peer mentoring events in schools nationwide.^31^, ^32^, ^33^ Additionally, the Women in Science, Technology, Engineering and Mathematics Ghana (WiSTEMGh) at the Kwame Nkrumah University of Science and Technology (KNUST) has conducted five editions of the STEM Girls’ Camp, targeting selected senior high schools in Ghana.^34^ These initiatives aim to raise awareness among students about study options, career paths, and research opportunities available within STEM disciplines.

There is a critical need to train the next generation of scientists while addressing the leaky pipeline to maintain interest in science. Children are more likely to envision themselves in scientific roles when they see individuals who look like them, whether in terms of race, ethnicity, sexual orientation, or gender.^21^ Strong female role models in math and science also boost confidence.^11^ Creating a gender‐inclusive STEM culture will harness diverse perspectives to solve future challenges. By inspiring confidence, showcasing role models, and discussing discrimination, we can empower girls to realize their potential in STEM fields.

In this study, we introduce high school students to the applications of medical physics, particularly in radiation oncology with the goal of a lifelong interest in science and technology. This outreach program utilizes interactive and hands‐on activities to create an engaging learning environment. The approaches, which incorporate higher‐level thinking skills, help students develop a genuine interest in physics. By demonstrating the practical applications of physics in healthcare, such as radiation oncology and diagnostic imaging, these programs can introduce students to a potentially rewarding career and contribute to the growth and inclusivity of the medical physics field.

This mini study describes a hands‐on educational activity designed to engage first‐year Senior High School science students at an all‐female school with the concept of a virtual patient simulation to treatment planning experience, all the while introducing them to concepts in radiation therapy and the unique role of professionals working together to provide quality care for patients. Additionally, this work is an extension of work done by Fagerstrom et al.^35^ for middle schoolers in the US.

Our work aligns with the Next Generation Science Standards (NGSS), which emphasize the importance of active learning and hands‐on experiences in science education.

## 2 METHODOLOGY

The study utilized a mixed‐methods approach, integrating both quantitative and qualitative data to assess the effectiveness of our “*heroes in radiation oncology*” program to high school students in Accra Ghana. This methodology allowed for a detailed understanding of student engagement, knowledge acquisition, and interest in STEM careers. Sixty‐two young girls in their first year in high school with ages ranging between 13 to 16 years participated in the program. Before the presentation, a pre‐assessment survey was distributed to the participating girls to evaluate their awareness of medical physics and radiation oncology. The survey aimed to establish baseline knowledge by gauging students' prior understanding of existing knowledge and attitudes toward these fields. The survey included questions about factors influencing students' career aspirations, such as curiosity in science, perceived job security, mentorship, financial incentives, and family guidance. Questions like financial incentives, and perceived job security were included in the pre‐assessment questionnaire because in the Ghanaian educational environment, the students avoid pursuing careers in the basic sciences because of the lack of availability of jobs and this is compounded in radiotherapy where only three radiotherapy centres are serving over 32 million population, this profession isn't visible at the high school level. Hence including these questions was to validate certain anecdotal cultural experiences

**FIGURE 1:**
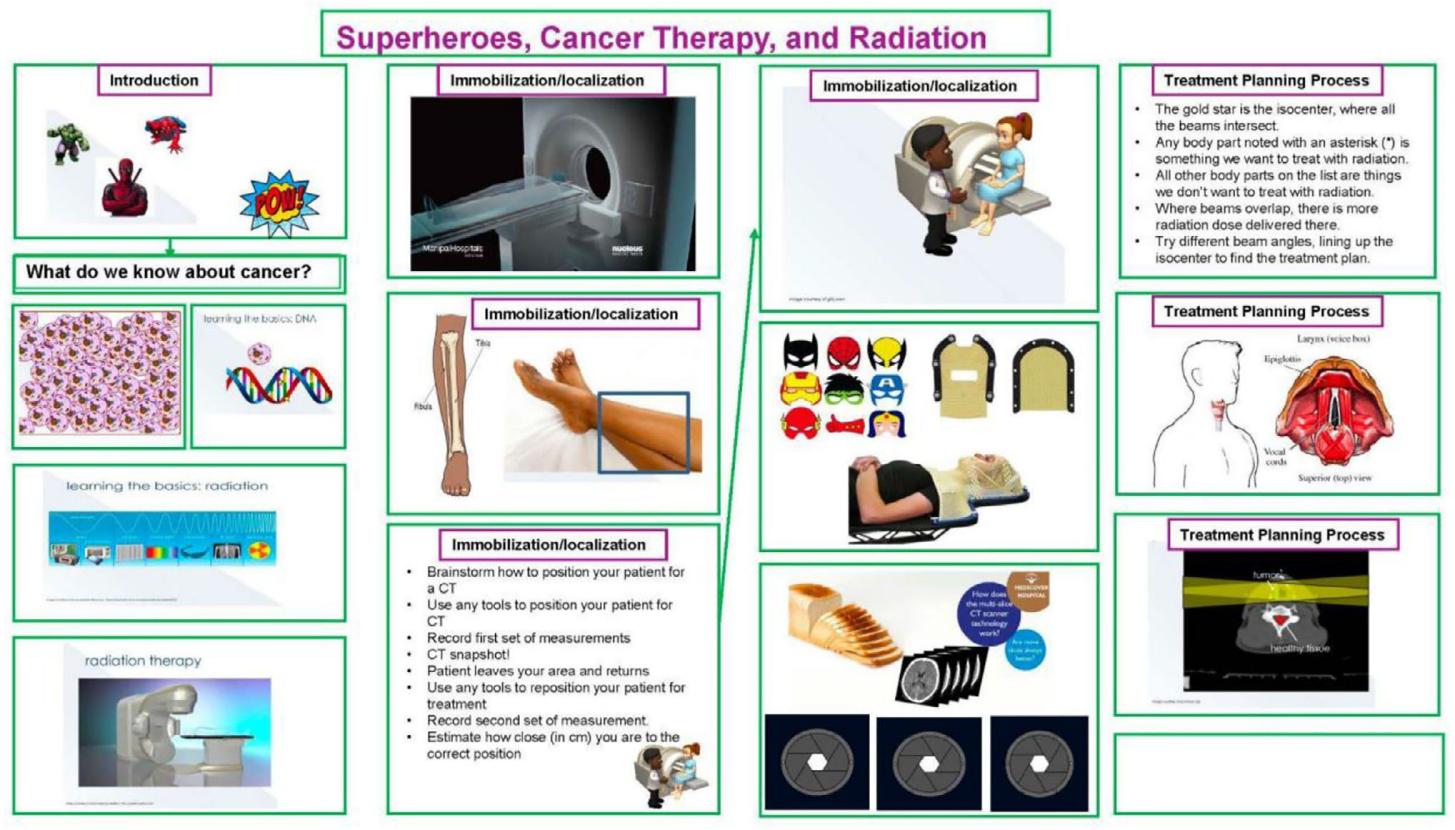
"Superheroes in radiation therapy" workshop designed for high school outreach programs, featuring educational modules on cancer biology, radiation therapy principles, immobilization techniques, treatment planning processes, and interactive demonstrations to engage students and foster interest in medical physics and oncology careers.

**FIGURE 2:**
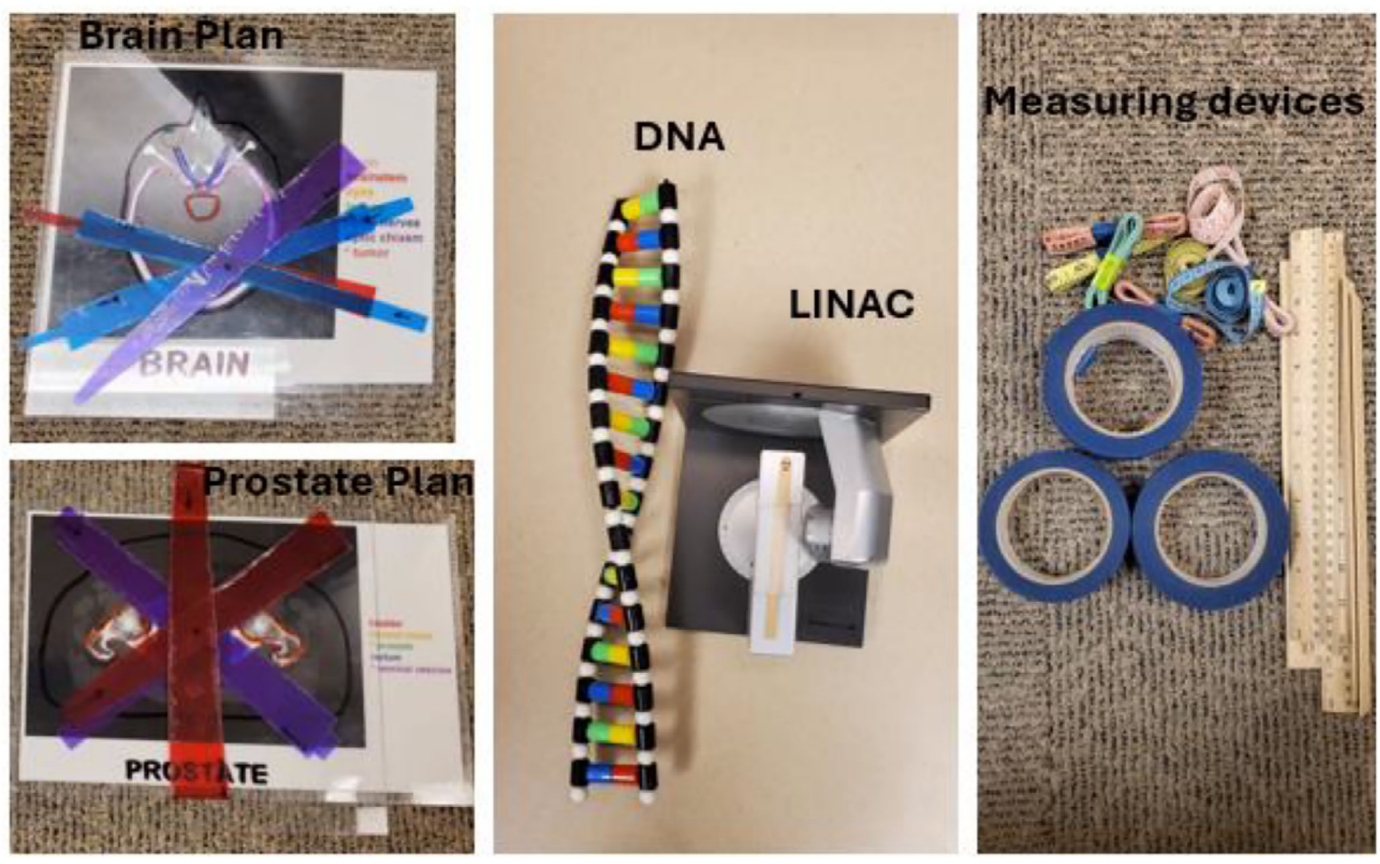
Illustrative items used in the “Superhero in radiotherapy” workshop, showcasing treatment plans (Brain and prostate) with labeled key organs and tumor locations (left),a model of DNA and a linear accelerator (LINAC) to demonstrate the interaction between radiation and biological systems (middle) and measuring devices and tapes for practicing positioning and re‐positioning (Right).

This was followed by an engaging session (See Figures 1 and 2) using relatable themes incorporating narratives about radiation, superheroes and Deoxyribonucleic Acid (DNA) mutations to simplify complex concepts. This approach aimed to capture students' interest and make scientific principles easily digestible. The use of mini linac models, plastic DNA, and head masks complemented the narrative, helping to illustrate the roles and responsibilities of the professionals in radiation oncology. After the presentation, students were divided into small groups of eight to participate in a hands‐on “from simulation to treatment planning activity” that reinforced the concepts discussed. A volunteer played the patient who underwent a patient pretend CT simulation set‐up, and the others in the group were tasked to record the set‐up position using tape measures, markers, and adhesive tapes. Using the recorded data students were encouraged to re‐set up the patient in an attempt to replicate the setup. Models of head masks were used to explain some of the immobilization devices used in radiotherapy.

This tactile experience helped demystify the technology and its applications. The student groups engaged in a hands‐on treatment planning activity utilizing treatment sheets of Two‐dimensional (2D) axial slice images of different anatomical sites with colored plastic cut out of divergent rays to aid students in creating three‐dimensional (3D) conformal plans. Each CT 2D axial slice had targets contoured with organs at risk (OAR) which were used to teach students the goal of every planner is to avoid. Plans created included the breast, prostate, lungs, and pancreas. The activities encouraged collaborative learning, as students worked together to problem solve and discuss concepts, enhancing their understanding through peer interaction.

After the activity session, post‐assessment surveys were collected to evaluate several key areas, including knowledge acquisition, knowledge about medical physics and radiation oncology, and students' interests in pursuing careers in the field. Understanding shifts in career aspirations provided insight into the program's impact. Our post‐assessment survey helped measure the effectiveness of the educational content. The students provided feedback on the presentation's effectiveness and the hands‐on activities, allowing evaluators to gauge the overall experience and areas for improvement.

## 3 RESULTS

### 3.1 Demographic

There were 65 participants, all girls aged 13 to 16 years. Each participant was in her first year of senior high school and enrolled in the general science program. Their elective subjects included biology, chemistry, physics, and mathematics, while their core subjects comprised Information Communication Technology (ICT), English, core mathematics, and social studies.

### 3.2 Pre‐presentation

Participants were asked about their career awareness and interests. 92.3% of the participants were aware of physician‐related careers, 83.1% were nursing, 73.8% were engineering, and 72.3% were research scientists. However, only 12.3% were aware of and interested in medical physics as a profession. Additionally, participants were asked about their current career interests. Using thematic analysis to analyze the responses and categorize them into

1. Healthcare professions (*Nursing*, *Doctor*, *Medical Doctor*, *Surgeon*, *Pediatrician*, *Dentist*, *Gynecologist*, *Neurologist*, *Pharmacist*, and *similar fields*).
2. Engineering and Technology (*Civil Engineer*, *Chemical Engineer*, *Biomedical Engineer*, *Computer Engineer*, *Petroleum Engineer*, and *Engineering* in general).
3. Science and Research (*Food Scientist*, *Biologist*, *Chemist*, *Psychologist*, and broader scientific aspirations like *Doctorate, Engineering, Scientist)*.
4. Multidisciplinary or Diverse Interests (*Law Scientist*, *Journalist*, *Robotic Engineer*, and *Legal Tech)*.
5. Uncertainty (Parental expectations affecting career choices).

### 3.3 Post‐presentation

**FIGURE 3:**
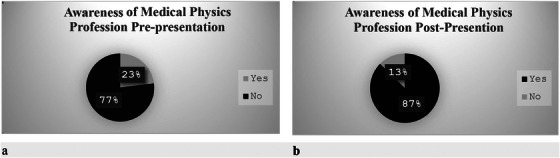
Change in participants' awareness of the medical physics profession before and after a presentation and hands‐on activities. Pre‐presentation, only 23% of participants were aware of the profession, which increased to 87% post‐presentation, demonstrating a significant impact of the outreach activity.

Figure 3 shows the change in students' perception of the medical physics profession before and after the superheroes in radiation therapy presentation. Additional feedback on students' perception of the field of medical physics was analysed using thematic analysis, and the response indicated that the presentation significantly improved participants’ awareness, understanding, and interest in medical physics. Additionally, it dispelled misconceptions about radiation safety and highlighted the interdisciplinary nature of the field and the career opportunities in medical physics. The thematic analysis presented in Table 1, underscores the success of the presentation in inspiring participants and expanding their perspectives on medical physics as s field of study. We encourage participants to ask questions and provide feedback to help us improve future educational outreach programs and to design a more sustainable program. Table 2 shows participants' questions categorized into themes.

**TABLE 1:** Thematic analysis of responses from participants after superheroes in radiotherapy workshop.

Theme	Description	Example quotes
**Increased awareness of medical physics**	Participants reported an enhanced understanding of the field.	“I didn't know physics could be used to diagnose diseases such as cancer.” “Did not know about such a profession and limited science professions but now, I have a further idea.”
**Career opportunities in medical physics**	Recognition of the diverse career paths in the field.	“There are more career opportunities in the physics background, not just being a physics teacher.” “That I can't only be a doctor if I learn physics, but I can be a radiation physicist.”
**Role of radiation in medical applications**	Highlighted knowledge about medical uses of radiation.	“Learning how radiation is used in CT scans reflected on a patient to see cancer cells in the body.” “Knowing how medical physics helps in curing cancers in all parts without hurting the healthy organ.”
**Changed perception of risk and safety**	Shifts in perceptions about the safety of working with radiation.	“It helped me understand that medical physics is not a risky work to do because the radiation worked with doesn't affect the workers.” “The radiation a person gets from eating a banana is more than the radiation someone gets from taking a CT scan once a year.”
**Increased interest in medical physics**	Growing interest in pursuing medical physics as a career or field of study.	“It has encouraged me to pursue medical physics.” “I have a better interest in medical physics.” “I thought it was difficult and kind of boring, but now I think it's interesting and cool.”
**Understanding of interdisciplinary nature**	Recognition of the integration of other sciences with physics in medical physics.	“I learnt that biology and math are also needed in their work.” “They need all other areas of science, including physics.”
**Practical applications in healthcare**	Interest in specific tools and functions, such as CT scans and radiotherapy.	“Learning about the CT scanner, how it looks, its functions and uses, really changed my life.” “Medical physics is a branch of science that helps patients get rid of tumours by radiation therapy.”
**Engagement and accessibility of the field**	The presentation made medical physics more approachable and engaging.	“It made me realize how interesting it was.” “I really enjoyed it and may just have to give physics a try in tertiary level.”

**TABLE 2:** Thematic analysis of participants feedback post interaction.

Theme	Description	Example quotes
**Career pathways and education**	Questions about how to pursue a career in medical physics, duration of study, and educational requirements.	“How long do we have to be in school for medical physics?” “Which courses in the university can you offer to become a medical physicist or radiotherapist?”
**Job opportunities**	Concerns about the availability and profitability of jobs in medical physics, especially in Ghana.	“Are scientists in Ghana more equipped in this field or is it few?” “Is the job opportunities under medical physics high in Ghana?”
**Safety and risks of radiation**	Concerns about potential health risks to professionals and patients related to radiation exposure.	“Don't the radiation harm patients?” “Is it deadly due to all the radiation a medical physicist is working with? Or does it shorten one's life span?”
**Practical applications and use cases**	Questions about the scope and applications of medical physics in diagnostics and treatment beyond cancer.	“Is radioactivity used in curing only cancer?”—“Can kidney problems be classified in medical physics?”
**Ethical and treatment outcomes**	Concerns about treatment success, patient recovery, and ethical challenges in radiotherapy.	‐ “If the patient receives treatment and still isn't recovering, what can be done?”—“Can a person still be cured if the tumor is at the secondary stage?”
**Radiation technology and techniques**	Questions about the functionality and safety of radiation‐related equipment and procedures.	“How are the radiation produced, and how useful are they?” “If during the operation of the CT scanner, something goes wrong, can it be reversed?”
**Integration with other fields**	Interest in combining medical physics with other disciplines, such as IT and engineering.	“While doing medical physics, can you still dive into IT and engineering?”
**Government and societal support**	Concerns about infrastructure, governmental support, and accessibility of radiotherapy in Ghana.	“Does the government show concern to medical physicians?” “Is radiotherapy actually applied in Ghana? It seems very expensive.”
**Interest and accessibility**	Requests for more resources and accessible knowledge about medical physics.	“Please can books be given out on more insights about medical physics? Because I am very interested in these careers.”

## 4 DISCUSSION

The GEM engaged a diverse group of 65 first‐year Senior High School girls, all enrolled in the general science program. This demographic is significant, as it represents an age for shaping career aspirations and interests. The initiative not only aimed to introduce students to medical physics but also sought to challenge cultural norms and perceptions that may deter young girls from pursuing careers in these areas.

### 4.1 Career awareness and interests

Pre‐presentation assessments revealed a significant disparity in career awareness among participants. While a high percentage expressed interest in traditional healthcare professions 92.3% for physician‐related careers and 83.1% for nursing only 12.3% were aware of medical physics as a viable career option. This stark contrast underscores a systemic issue in the representation of medical physics within the educational and career guidance available. The categorization of career interests into themes as healthcare, engineering and science highlights the avenues to which students envision their futures. The low awareness of medical physics stems from factors such as cultural perceptions, a lack of exposure to role models in the field. The thematic analysis revealed influences by parental expectations, suggesting that family dynamics significantly shape students' career choices. Traditional gender roles and societal expectations can limit young girl's aspirations, particularly in fields perceived as male‐dominated. This indicates a need for targeted initiatives that not only promote medical physics but also address the cultural narratives that may discourage young girls from pursuing unconventional paths in STEM.

### 4.2 Impact of the presentation

The post‐presentation analysis showed a significant shift in the students' perceptions of medical physics. The interactive and engaging format of the “superheroes in radiation therapy” presentation increased awareness and interest in the field. Participants reported a newfound understanding of the interdisciplinary nature of medical physics, its connections to biology, mathematics, and engineering. The approach allowed them to appreciate how various scientific disciplines converge in the realm of medical physics. The presentation dispelled misconceptions regarding radiation safety. Participants initially viewed radiation‐related careers as risky; however, they left with an understanding of how medical physicists function. This shift in perception counters the stigma surrounding careers in radiation and the importance of informed education in fostering interest in STEM fields. The activities, which included patient simulations and treatment planning, not only reinforced theoretical concepts but also provided practical experience that is often lacking in traditional classroom settings. By engaging in these activities, students developed critical thinking and problem‐solving skills, essential competencies in any scientific career. These exercises fostered teamwork and communication skills that are invaluable in both academic and professional settings.

### 4.3 Long‐term implications

The success of the initiative suggests that similar programs could help in bridging the gender gap in STEM, particularly in medical physics. The feedback collected regarding their interest in medical physics indicates a positive trajectory for future enrollment. The program's alignment with the NGSS emphasizes the importance of active learning and engagement in science education. To maximize the impact of such initiatives, connecting students with female role models in medical physics and related fields can provide guidance and encouragement, helping to sustain their interest and commitment to pursuing STEM careers. Incorporating follow‐up activities or workshops can reinforce learned concepts and facilitate deeper exploration into medical physics and its applications.

This discussion expands on the findings from Table 1 and integrates the themes from Table 2 to provide an understanding of the program's impact.

1. **Increased Awareness of Medical Physics**: The program raised awareness of medical physics among participants, many of whom were previously unfamiliar with the field. As evidenced by quotes like, “I didn't know physics could be used to diagnose diseases such as cancer,” the outreach initiative opened doors to discussions on the importance of medical physics in modern medicine. The subsequent questions regarding how to pursue a career in medical physics, such as “How long do we have to be in school for medical physics?” reflect a newfound curiosity and willingness to explore this pathway.
2. **Career Opportunities in Medical Physics**: Participants recognized the diverse career opportunities available within medical physics. Remarks like, “There are more career opportunities in the physics background, not just being a physics teacher,” indicate a shift in mindset. The concerns about job availability and profitability in the Ghanaian context emerged. Questions such as, “Are scientists in Ghana more equipped in this field or is it few?” and “Is the job opportunities under medical physics high in Ghana?” reveal the need for clearer information regarding career prospects. Addressing these concerns is essential for fostering sustained interest among students, as understanding the job market can significantly influence their career decisions.
3. **Role of Radiation in Medical Applications**: The program enhanced participants' understanding of the role of radiation in medical applications. Students expressed newfound knowledge about how radiation is utilized in diagnostics and treatment, with quotes like, “Learning how radiation is used in CT scans reflected on a patient to see cancer cells in the body.” Questions such as, “Don't the radiation harm patients?” and “Is it deadly due to all the radiation a medical physicist is working with?” indicate that, despite increased awareness, misconceptions about radiation safety persist. This highlights the need for ongoing education to address these fears and reinforce the safety measures in place within the profession.
4. **Changed Perception of Risk and Safety**: Participants had a shift in their perceptions of the safety of working with radiation. Through education, they began to understand that, when managed properly, radiation is safe for both professionals and patients. Questions about the functionality and safety of radiation‐related equipment, such as, “How the radiation are produced, and how useful are they?” emphasize the need for comprehensive educational resources that clarify these technical aspects. By addressing these questions, educators can help demystify the technology and enhance students' confidence in pursuing careers in medical physics.
5. **Government and Societal Support**: Participants expressed concerns about the infrastructure and governmental support for medical physics in Ghana. Questions such as, “Does the government show concern to medical physicists?” and “Is radiotherapy actually applied in Ghana? It seems very expensive,” reveal anxieties regarding the accessibility of medical physics services in their country. These concerns underscore the importance of societal support and investment in healthcare infrastructure to ensure that future medical physicists can practice effectively and contribute to improving patient outcomes.

## CONFLICT OF INTEREST STATEMENT

The authors declare no conflicts of interest.
